# Domestic and urban violence faced by community health workers: a multidimensional analysis in vulnerable territories in northeastern Brazil during and after the COVID-19 pandemic

**DOI:** 10.1016/j.lana.2026.101436

**Published:** 2026-03-11

**Authors:** Marcella R. Cardoso, Maria Cecília Ramiro Talarico, Roger Silva Sousa, Sidney Feitosa Farias, Franklin Delano Forte, Yana Paula Coelho Correia Sampaio, Ana Patrícia Pereira Morais, Mary Greenwald, Marcia C. Castro, Aisha K. Yousafzai, Annekathryn Goodman, Anya Pimentel Gomes Fernandes Vieira-Meyer

**Affiliations:** a Department of Obstetrics and Gynecology, Division of Gynecologic Oncology, Massachusetts General Hospital, Boston, MA, USA; b Harvard Medical School, Massachusetts General Hospital, Boston, MA, USA; c Center for Global Health, Massachusetts General Hospital, Boston, MA, USA; d Department of Obstetrics and Gynecology, School of Medical Sciences, Universidade Estadual de Campinas - UNICAMP, Campinas, SP, Brazil; e Fundação Oswaldo Cruz Pernambuco, Recife, PE, Brazil; f Universidade Federal da Paraíba, João Pessoa, PB, Brazil; g Faculdade de Medicina Estácio de Juazeiro do Norte, Juazeiro do Norte, CE, Brazil; h Universidade Estadual do Ceará, Fortaleza, CE, Brazil; i Harvard T.H. Chan School of Public Health, Boston, MA, USA; j Fundação Oswaldo Cruz Ceará, Eusébio, CE, Brazil

**Keywords:** Violence against women, Gender-based violence, Brazil, Homicide, Public health

## Abstract

**Background:**

Community Health Workers (CHWs) play a critical role in frontline public health, especially in vulnerable regions. However, their exposure to violence remains an understudied issue. This study explores how CHWs gender shape the perception and experience of different types of violence in Northeastern Brazil during and after the COVID-19 pandemic.

**Methods:**

We conducted a cross-sectional analysis using data collected from 3800 CHWs in 2021 and 2023, across eight municipalities in Northeastern Brazil. Data were analyzed by gender, comparing perceptions and reported experiences of domestic (DV) and urban violence (UV). Inference used Cochran-Mantel-Haenszel (CMH) pooled odds ratios with Breslow–Day tests for homogeneity.

**Findings:**

Male CHWs consistently reported higher perception to urban violence (CMH OR 0.67 [95% CI 0.53–0.83]; *p* < 0.001), while female CHWs reported greater awareness of domestic violence (CMH OR 1.18 [1.00–1.40]; *p* = 0.058). Direct victimization was higher among males, particularly for physical aggression (CMH OR 0.80 [0.67–0.97]; *p* = 0.023), stabbing (CMH OR 0.75 [0.59–0.95]; *p* = 0.022), and non-lethal gunshot incidents (CMH OR 0.71 [0.58–0.87]; *p* < 0.001). In contrast, females consistently reported higher awareness of rape across both years (CMH OR 1.40 [1.15–1.70]; *p* < 0.001).

**Interpretation:**

Violence is not experienced or perceived uniformly, rather, it is shaped by gender roles and context. Female CHWs demonstrated greater sensitivity to domestic and sexual violence, while male CHWs were more attuned to urban violence and criminal dynamics. These are self-reported signals and should be interpreted with appropriate caution, yet they provide actionable intelligence for local prevention. Recognizing CHWs as strategic observers of community suffering, this study underscores the urgent need for intersectional, gender-responsive, and territorially differentiated public policies.

**Funding:**

This project was funded by 10.13039/501100005283FUNCAP, Fiocruz-PMA, and the Lemann Research Fund from 10.13039/100007229Harvard University.

## Resumo

**Contexto:**

As Agentes Comunitárias de Saúde (ACS) desempenham um papel fundamental na linha de frente da saúde pública, especialmente em regiões vulneráveis. No entanto, sua exposição à violência permanece uma questão pouco estudada. Este estudo explora como o gênero dos ACS molda a percepção e a experiência de diferentes tipos de violência no Nordeste do Brasil durante e após a pandemia de COVID-19.

**Métodos:**

Realizamos uma análise transversal utilizando dados coletados de 3.800 ACS em 2021 e 2023, em oito municípios do Nordeste do Brasil. Os dados foram analisados por gênero, comparando percepções e experiências relatadas de violência doméstica (VD) e violência urbana (VU). As inferências utilizaram odds ratios combinados de Cochran–Mantel–Haenszel (CMH) com testes de Breslow–Day para homogeneidade.

**Achados:**

ACS do gênero masculino relataram consistentemente maior percepção de violência urbana (OR CMH 0,67 [IC 95% 0,53–0,83]; p < 0,001), enquanto ACS do gênero feminino relataram maior consciência de violência doméstica (OR CMH 1,18 [1,00–1,40]; p = 0,058). A vitimização direta foi maior entre homens, especialmente para agressão física (OR CMH 0,80 [0,67–0,97]; p = 0,023), esfaqueamento (OR CMH 0,75 [0,59–0,95]; p = 0,022) e episódios de ferimento por arma de fogo não letal (OR CMH 0,71 [0,58–0,87]; p < 0,001). Em contraste, mulheres relataram consistentemente maior conhecimento de casos de estupro em ambos os anos (OR CMH 1,40 [1,15–1,70]; p < 0,001).

**Interpretação:**

A violência não é vivenciada ou percebida de forma uniforme; ela é moldada por papéis de gênero e pelo contexto. ACS mulheres demonstraram maior sensibilidade à violência doméstica e sexual, enquanto ACS homens estiveram mais atentos à violência urbana e às dinâmicas criminais. Esses são sinais autorrelatados e devem ser interpretados com a devida cautela, mas fornecem inteligência acionável para prevenção local. Ao reconhecer os ACS como observadores estratégicos do sofrimento comunitário, este estudo reforça a necessidade urgente de políticas públicas interseccionais, sensíveis ao gênero e territorialmente diferenciadas.

**Financiamento:**

Este projeto foi financiado pela FUNCAP, Fiocruz-PMA e pelo Lemann Research Fund da Universidade Harvard.

**Research in context**

**Evidence before this study**

Before conducting this study, we performed a comprehensive review of peer-reviewed literature using PubMed, Scopus, SciELO, and Google Scholar. Our search spanned publications from January 2020 to March 2025 and included articles in English, Portuguese, and Spanish. We used combinations of the following terms: “community health workers”, “violence perception”, “gender-based violence”, “urban violence”, “COVID-19”, “Brazil”, and “territorial inequality.” Studies were included if they addressed community health workers' (CHWs') experiences or perceptions of violence, particularly in relation to the pandemic, and explored gender or geographic disparities. We excluded studies that did not focus directly on CHWs or lacked an intersectional perspective. The literature revealed very few studies assessing CHWs' dual roles as both health professionals and community members navigating violence, especially in Latin America. Most existing research was concentrated in large metropolitan areas and rarely disaggregated data by gender. We found no published studies that simultaneously analyzed CHWs’ perceptions of domestic and urban violence through the combined lenses of gender and time. This highlights a significant gap in understanding how frontline health workers perceive and respond to violence in different social settings.

**Added value of this study**

This study contributes novel evidence from Brazil and adds to a small body of international work by examining how gender and time intersect to shape CHWs' perceptions of domestic and urban violence. While previous research has explored violence during the COVID-19 pandemic, peer-reviewed studies rarely disaggregate CHW experiences by gender across both pandemic and post-pandemic periods. By applying this multidimensional lens, our findings reveal how gender influences not only the types of violence perceived but also the ability to recognize and respond to the conditions created by violence. Female CHWs more often perceived domestic violence and showed higher sentinel awareness of sexual violence, while male CHWs more often identified threats linked to territorial and organized crime. This study brings new evidence on how CHWs, as trusted frontline providers embedded in the communities they serve, are often the first to become aware of cases of gender-based violence, yet they lack institutional support, training, and clear protocols to act. While the return to in-person visits revealed a surge in previously hidden cases of violence, particularly in marginalized territories, many CHWs did not feel empowered or protected to intervene, and some questioned whether responding to violence should fall within their professional role. Our findings highlight the urgent need to develop gender-sensitive and territory-based strategies that clarify the scope of CHWs’ responsibilities and provide safe, structured channels for action, respecting both their role in public health and the constraints of their lived realities.

**Implications of all the available evidence**

Taken together, the available evidence underscores the urgent need for integrated strategies that both strengthen community safety and protect CHWs themselves. As frontline actors embedded in vulnerable territories, CHWs are uniquely positioned to identify emerging patterns of urban and domestic violence. However, without institutional guidance, adequate training, and protective measures, their ability to act remains constrained, and their own well-being jeopardized. Our findings suggest that future policies must move beyond broad statements about gender sensitivity responsiveness, and instead prioritize concrete, actionable mechanisms. These include standardized training to help CHWs recognize and respond to different forms of violence including subtle psychological abuse and the integration of CHW reporting into local and national surveillance systems to ensure systematic data flows from communities to health and protection sectors. They also include robust referral pathways and intersectoral networks linking survivors to legal, social, and mental health services, alongside structural protections for CHWs themselves safety protocols, mental health support, and fair remuneration that acknowledges their dual exposure as professionals and community members. By embedding these strategies into health and social policy, Brazil, and other countries facing similar challenges, can leverage CHWs’ proximity to vulnerable populations while reducing the risks of burnout, retraumatization, and insecurity. Far from being passive witnesses, CHWs represent a critical human infrastructure for violence surveillance, prevention, and response, and ensuring their protection and empowerment is therefore not only a matter of worker rights, but also a public health and social justice imperative.

## Introduction

Violence is widely recognized as a serious public health problem with profound impacts on individuals and communities. Globally, the burden of violence is enormous: an estimated one in three women (around 736 million worldwide) have experienced physical and/or sexual violence in their lifetime.^1^ Furthermore, many societies also grapple with high levels of urban violence and organized crime. For instance, in 2019, injuries and violence were responsible for approximately 4.4 million deaths globally.^2^

Gender-based violence (GBV) remains a persistent and deeply critical issue in Brazil, which consistently ranks among the countries with the highest femicide rates.^3^ Structural inequalities, such as low income, racial marginalization, and weak institutional protections, contribute to heightened vulnerability to violence in certain communities.^3^ This form of violence is deeply rooted in patriarchal norms and gender inequalities, and tends to be most pronounced in socioeconomically disadvantaged settings, such as many areas of Northeastern Brazil, with low human development indices and persistent gender inequities.^4^^,^^5^ These same structural conditions also contribute to high levels of urban violence in Brazil, including assaults, armed confrontations, and gang activity, which remain a persistent reality, especially in urban and peri-urban areas marked by social inequality, historical exclusion, and limited State presence, where vulnerable populations face heightened risk of exposure to violence.^6^

Within this context, community health workers (CHWs) play a pivotal role at the frontlines of Brazil's public healthcare system and are uniquely positioned to confront the issue of violence, as they live and work in the same territory as the population they serve and are exposed to similar socio-environmental factors. Incorporated into the primary care teams of the Family Health Strategy, the cornerstone of Brazil's Unified Health System (SUS), CHWs serve as a vital link between formal health services and the community.^7^ Although CHWs are often among the first to observe or learn of cases of domestic and community violence, there is limited research systematically examining how they encounter violence, whether through direct victimization, witnessing, or second-hand reports, and how these experiences shape their ability to deliver care, maintain their own well-being, and contribute to community-level surveillance and response. The COVID-19 pandemic has thrown into sharp view both the crucial importance of CHWs and the heightened challenges they face in contexts of violence.^8^^,^^9^

Lockdown measures, economic hardship, and prolonged confinement at home led to an increase in domestic violence globally, a phenomenon widely described as a “shadow pandemic” of COVID-19,^10^ with spikes of 25–35% in intimate partner violence increase in multiple countries.^11^ In Brazil, early evidence suggested a significant surge in gender-based violence and femicides during the pandemic. One analysis linked the implementation of physical distancing measures to a 50% increase in reported domestic violence cases in some regions of Brazil.^12^ Concurrently, many Brazilian cities experienced shifts in community violence dynamics; some saw temporary declines in certain crimes during lockdown, while others later experienced upticks in gang-related violence.^13^ These pandemic-driven changes created new challenges for CHWs.

There remains a significant gap in the literature regarding how CHWs, especially those operating in Brazil's high-risk territories, perceive and are affected by violence in their day-to-day work. Few studies investigate how CHWs, as both healthcare providers and community members, experience violence in vulnerable territories. Particularly in Latin America, there is a scarcity of data on how CHWs navigate their dual role as healthcare providers and community members amidst endemic violence. Even fewer studies have adopted a temporal lens that captures how these patterns may have shifted during and after the COVID-19 pandemic. These gaps highlight a public health concern with global implications. The convergence of GBV, community-level vulnerability, and the lingering impact of the pandemic underscores the urgent need for multidimensional approaches that go beyond clinical care. To this end, the study addresses two main questions: (1) how patterns of violence differ by gender among CHWs and (2) how reported violence perception and experiences differ between 2021 (during the pandemic) and 2023 (post-pandemic). Together, these objectives provide a comprehensive framework to understand the role of gender, in shaping CHWs' experiences of violence.

## Methods

### Design and data source

**Fig. 1:**
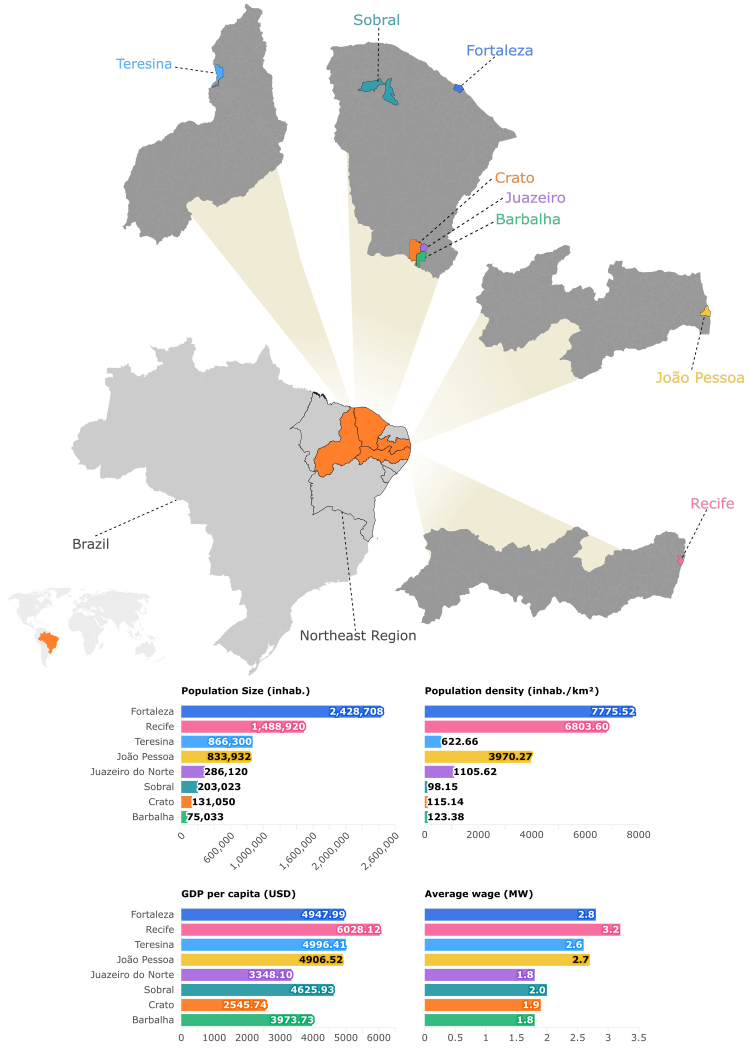
Geographic location and demographic and socioeconomic indicators of the municipalities included in the study. The figure shows the geographic distribution of eight municipalities in the Northeast Region of Brazil, including four state capitals (Fortaleza, Recife, Teresina, and João Pessoa) and four non-capital cities (Juazeiro do Norte, Crato, Barbalha, and Sobral). Comparative charts based on the most recent official data available (2021–2022). Inhab: inhabitants. GDP: Gross Domestic Product. USD: United States Dollar. MW: Minimum Wage.

The reporting of this study follows the STROBE guidelines for cross-sectional studies. This is a multicenter study with a quantitative approach, conducted in two periods of time (2021 and 2023) within the scope of Primary Health Care (PHC) in various municipalities of Northeastern Brazil. The study included four state capitals: Fortaleza (Ceará), João Pessoa (Paraíba), Recife (Pernambuco), and Teresina (Piauí), as well as four (non-capital) inner-cities of Ceará state: Crato, Juazeiro do Norte, Barbalha, and Sobral. The geographic distribution and key demographic indicators of these municipalities are illustrated in Fig. 1.

The study involved CHWs who were randomly selected and invited to participate, provided they worked and resided in the same neighborhood and had been in service for at least one year. CHWs who were on vacation or medical leave during either of the data collection periods were excluded. A simple random sampling method was used to calculate the sample size for both collections (2021 and 2023), adopting a 5% margin of error, a 95% confidence level, and an assumed population distribution of 80/20. The sample size was calculated for each municipality based on the CHW population registered in the e-Gestor platform (Ministry of Health) in 2020 (N = 7909) and in 2022 (N = 7660). The resulting sample sizes were 1942 CHWs in 2021 and 1907 in 2023. High participation was observed, with an estimated non-response rate of less than 5% in both data collection periods. The few refusals were primarily due to lack of time to complete the full questionnaire rather than discomfort with the study topic. Given this low level of non-participation and the absence of systematic patterns in refusals, the potential for selection bias due to non-response is considered minimal.

The data analyzed in this manuscript derive from a broader multicentric study on CHWs work process, health, COVID-19, and urban violence in Northeastern Brazil. For the purposes of this manuscript, we conducted a focused secondary analysis examining patterns of violence, stratified by CHWs gender and survey year (2021 and 2023). Gender was self-reported with response options “feminine”, “masculine”, and an open “other” category with free-text specification. Data collection took place from April 1 to August 31, 2021, and from July 3 to November 30, 2023. Data collection in 2021 represented the earliest moment in which in-person fieldwork could be safely resumed after the strict COVID-19 restrictions that prevented any primary data collection after the pandemic onset. A second wave in 2023 was intentionally conducted following the WHO declaration that COVID-19 was no longer a Public Health Emergency of International Concern, allowing us to document CHWs’ perceptions of violence under a post-pandemic context using the same sampling frame and instruments. These two timepoints, therefore, capture conditions during and after the acute pandemic phase. Table 1 summarizes the sociodemographic characteristics of respondents by gender and year.

**Table 1:** Sociodemographic characteristics of community health workers, by gender and year (2021 and 2023).

Characteristic	2021			2023		
	Female	Male	Total	Female	Male	Total
	N = 1599^a^	N = 343^a^	N = 1942^a^	N = 1554^a^	N = 353^a^	N = 1907^a^
**Age**						
<40	348 (22%)	119 (35%)	467 (24%)	236 (16%)	105 (31%)	341 (19%)
40–60	1137 (72%)	211 (62%)	1348 (70%)	1102 (74%)	228 (66%)	1330 (73%)
>60	92 (6%)	9 (3%)	101 (6%)	145 (10%)	10 (3%)	155 (8%)
Unknown	22	4	26	71	10	81
**Race**						
White	207 (13%)	38 (11%)	245 (13%)	191 (12%)	39 (11%)	230 (12%)
Brown	1148 (72%)	247 (72%)	1395 (72%)	1113 (72%)	246 (70%)	1359 (71%)
Black	235 (15%)	57 (17%)	292 (15%)	223 (14%)	58 (16%)	281 (15%)
Other	6 (0.4%)	1 (0.3)	7 (0.4%)	20 (1%)	6 (2%)	26 (1%)
Unknown	3	0	3	7	4	11
**Domestic Partner**						
Yes	932 (58%)	201 (59%)	1133 (58%)	867 (56%)	210 (59%)	1077 (56%)
No	667 (42%)	142 (41%)	809 (42%)	683 (44%)	141 (40%)	824 (43%)
Unknown	–	–	–	4	2	6
**Education**						
High School Incomplete	144 (9%)	17 (5%)	161 (8%)	109 (7%)	14 (4%)	123 (6%)
High School	766 (48%)	138 (40%)	904 (47%)	755 (49%)	130 (37%)	885 (46%)
College degree or in progress	689 (43%)	188 (55%)	877 (45%)	685 (44%)	206 (58%)	891 (47%)
Unknown	–	–	–	5	3	8
**Years as CHW**						
<5	55 (3%)	19 (6%)	74 (4%)	47 (3%)	20 (6%)	67 (4%)
5–10	177 (11%)	88 (26%)	265 (14%)	101 (7%)	47 (15%)	148 (9%)
>10	1352 (85%)	233 (69%)	1585 (82%)	1272 (90%)	244 (78%)	1516 (88%)
Unknown	15	3	18	134	42	176

a n (%).

For both data collection periods, the research team was trained in all participating municipalities. Training included administration of data collection instruments, COVID-19 safety protocols, and role-play simulation techniques. A random selection of CHWs was made based on information given by municipal health secretariats (names of CHW linked to each PHC health unit in the municipality).

The selection of municipalities, four state capitals and four inner-cities in Northeastern Brazil, reflected their classification as high-vulnerability and high-violence territories, based on national epidemiological indicators and longstanding relevance to territorial health surveillance. These settings were part of the broader multicentric study from which the present analysis derives, and together they represent diverse urban and socioeconomic contexts where CHWs face elevated risk of exposure to violence.

Data were collected using a structured questionnaire (Supplementary Material) which included socioeconomical information, work-related questions, and items assessing CHWs’ exposure to and perception of violence within the communities where they live and work. First, we ask about their perception of general violence, if violence is present in their community. Then, for analytical purposes, we distinguish between urban violence (UV), defined as violence occurring in public or community spaces, such as assaults, robberies, or gang conflicts and domestic violence (DV), defined as violence within the household, including intimate partner and intra-family abuse.

Exposure to violence was examined across three dimensions: (*i*) perception of violence, reflecting how CHWs interpret and make sense of violence in their territories (Table 2–General Violence, Urban Violence and Domestic Violence); (*ii*) direct experience, referring to cases where CHWs themselves (or their immediate family) were victims (Table 3–Suffered); and (*iii*) witnessed or learned violence, encompassing incidents they observed or were informed of through their community networks (Table 3–Heard about/saw). Although these dimensions are interconnected, they capture different aspects of how violence is manifested and understood in CHWs’ daily lives and professional practice. These distinctions guided the analyses presented in Tables 2 and 3.

**Table 2:** Perception of the presence and types of violence in the communities served by Community Health Workers (CHWs), by gender and year (2021 and 2023).

Variables	2021				2023				2021 & 2023		
	Female	Male	Total	*p*-value^b^	Female	Male	Total	*p*-value^b^	CMH OR 95%	CMH *p*-value	BD *p*-value
	N = 1599^a^	N = 343^a^	N = 1942^a^		N = 1554^a^	N = 353^a^	N = 1907^a^				
**General violence**											
Yes	1205 (76%)	271 (79%)	1476 (76%)	0.20	1198 (78%)	282 (80%)	1480 (78%)	0.30	0.84 (0.69–1.03)	0.11	0.87
No	381 (24%)	71 (21%)	452 (23%)		342 (22%)	69 (20%)	411 (22%)				
Missing	13	1	14		14	2	16				
**Domestic violence**											
Yes	737 (48%)	152 (46%)	889 (48%)	0.50	705 (45%)	139 (39%)	844 (44%)	0.041	1.18 (1.00–1.40)	0.06	0.34
No	794 (52%)	178 (54%)	972 (52%)		849 (55%)	214 (61%)	1063 (56%)				
Missing	68	13	81		0	0	0				
**Urban violence**											
Yes	1212 (79%)	280 (85%)	1492 (80%)	0.019	1223 (79%)	300 (85%)	1523 (80%)	0.008	0.67 (0.53–0.83)	<0.001	0.87
No	319 (21%)	50 (15%)	369 (20%)		331 (21%)	53 (15%)	384 (20%)				
Missing	68	13	81		0	0	0				

CMH: Cochran-Mantel-Haenszel test; BD: Breslow–Day test.

General violence refers to CHWs perception of violence in their community. Urban violence refers to assaults, armed conflicts, gang-related activities, and other forms of violence occurring in public spaces and community settings. Domestic violence refers exclusively to violence within households or intimate relationships.

a n (%).

b Chi-square test.

**Table 3:** CHWs’ exposure and awareness of violence by gender over time.

Types of violence	2021				2023				2021 & 2023		
	Female	Male	Total	*p*-value^b^	Female	Male	Total	*p*-value^b^	CMH OR 95%	CMH *p*-value	BD *p*-value
	N = 1599^a^	N = 343^a^	N = 1942^a^		N = 1554^a^	N = 353^a^	N = 1907^a^				
**Heard about/Saw**											
**Physical aggression**											
Yes	1013 (64%)	215 (63%)	1228 (64%)	0.80	975 (75%)	226 (78%)	1201 (75%)	0.20	0.95 (0.78–1.14)	0.61	0.27
No	575 (36%)	126 (37%)	701 (36%)		328 (25%)	63 (22%)	391 (25%)				
Missing	11	2	13		251	64	315				
**Assault**											
Yes	1178 (74%)	256 (76%)	1434 (75%)	0.60	1029 (81%)	247 (88%)	1276 (83%)	0.014	0.80 (0.64–1.00)	0.06	0.09
No	406 (26%)	82 (24%)	488 (25%)		234 (19%)	35 (12%)	269 (17%)				
Missing	15	5	20		291	71	362				
**Stabbing**											
Yes	539 (34%)	101 (30%)	640 (33%)	0.12	584 (45%)	122 (42%)	706 (44%)	0.40	1.17 (0.98–1.41)	0.09	0.66
No	1040 (66%)	238 (70%)	1278 (67%)		715 (55%)	168 (58%)	883 (56%)				
Missing	20	4	24		255	63	318				
**Non-lethal gunshot**											
Yes	838 (53%)	175 (52%)	1013 (53%)	0.70	779 (61%)	179 (63%)	958 (61%)	0.40	0.98 (0.82–1.17)	0.87	0.39
No	743 (47%)	163 (48%)	906 (47%)		508 (39%)	105 (37%)	613 (39%)				
Missing	18	5	23		267	69	336				
**Rape**											
Yes	449 (28%)	74 (22%)	523 (27%)	0.016	499 (39%)	91 (31%)	590 (37%)	0.019	1.40 (1.15–1.70)	<0.001	0.93
No	1129 (72%)	262 (78%)	1391 (73%)		789 (61%)	199 (69%)	988 (63%)				
Missing	21	7	28		266	63	329				
**Gang violence**											
Yes	815 (51%)	184 (54%)	999 (52%)	0.30	788 (62%)	193 (67%)	981 (63%)	0.09	0.84 (0.71–1.01)	0.06	0.55
No	772 (49%)	154 (46%)	926 (48%)		485 (38%)	94 (33%)	579 (37%)				
Missing	12	5	17		281	66	347				
**Suffered**											
**Physical aggression**											
Yes	544 (35%)	126 (39%)	670 (36%)	0.20	384 (35%)	104 (42%)	488 (36%)	0.027	0.80 (0.67–0.97)	0.023	0.37
No	989 (65%)	198 (61%)	1187 (64%)		715 (65%)	141 (58%)	856 (64%)				
Missing	66	19	85		455	108	563				
**Assault**											
Yes	920 (59%)	197 (61%)	1117 (59%)	0.60	701 (60%)	165 (63%)	866 (60%)	0.30	0.90 (0.75–1.08)	0.30	0.68
No	635 (41%)	127 (39%)	762 (41%)		472 (40%)	96 (37%)	568 (40%)				
Missing	44	19	63		381	92	473				
**Stabbing**											
Yes	205 (13%)	51 (16%)	256 (14%)	0.30	173 (16%)	54 (22%)	227 (17%)	0.023	0.75 (0.59–0.95)	0.022	0.38
No	1321 (87%)	273 (84%)	1594 (86%)		906 (84%)	190 (78%)	1096 (83%)				
Missing	73	19	92		475	109	584				
**Non-lethal gunshot**											
Yes	371 (24%)	89 (28%)	460 (25%)	0.20	247 (22%)	84 (34%)	331 (25%)	<0.001	0.71 (0.58–0.87)	<0.001	0.06
No	1164 (76%)	234 (72%)	1398 (75%)		852 (78%)	166 (66%)	1018 (75%)				
Missing	64	20	84		455	103	558				
**Rape**											
Yes	169 (11%)	40 (12%)	209 (11%)	0.50	155 (14%)	34 (14%)	189 (14%)	0.80	0.96 (0.73–1.26)	0.81	0.55
No	1358 (89%)	285 (88%)	1643 (89%)		919 (86%)	211 (86%)	1130 (86%)				
Missing	72	18	90		480	108	588				
**Gang violence**											
Yes	363 (24%)	87 (27%)	450 (24%)	0.20	290 (26%)	93 (38%)	383 (28%)	<0.001	0.84 (0.71–1.01)	0.06	0.55
No	1168 (76%)	238 (73%)	1406 (76%)		820 (74%)	155 (63%)	975 (72%)				
Missing	68	18	86		444	105	549				

Proportion of CHWs who witnessed or experienced violence in their area of practice, personally or through a family member living in the same area.

CMH: Cochran-Mantel-Haenszel test; BD: Breslow–Day test.

Heard about/Saw refers to cases in which the CHW witnessed or learned about the occurrence of that type of violence affecting others in their community (indirect exposure). Suffered refers to cases in which the CHW personally (or immediately family) experienced the specified form of violence (direct exposure).

Only affirmative (“yes”) responses were included. Multiple types of violence could be reported per respondent.

a n (%).

b Pearson's Chi-squared test.

### Statistical analysis

In the present study, CHWs who live and work in the same community were included (1942 in 2021 and 1907 in 2023). To characterize the sample, descriptive analyses were conducted, including the calculation of absolute and relative frequencies for categorical variables. Comparative analyses between different periods and/or study settings were performed using Pearson's chi-squared test with simulated *p-*values (based on 2000 replicates), to account for distributional assumptions.

The primary comparison was gender (female *vs* male) stratified by year (2021 and 2023). Dichotomous variables (items with a “yes” response) were analyzed using 2 × 2 tables within each year and then combined via the Cochran-Mantel-Haenszel (CMH) method to estimate a common odds ratio (OR) with a 95% CI and the CMH chi-square test (*p*-value). Homogeneity of stratum-specific ORs (years) was assessed with the Breslow–Day (BD) test; BD *p* > 0.05 was interpreted as supporting the reporting of the pooled CMH OR. For within-year descriptions, we used Pearson's chi-square test (female *vs* male). The analysis was applied to perception outcomes (general violence, domestic violence, urban violence) and to direct victimization and indirect exposure specific items (physical aggression, assault/robbery, stabbing, non-lethal gunshot, rape, gang violence). Multiple types of violence could be reported per respondent. Missing responses were treated as missing, not as negative answers. We used two-sided tests with α = 0.05 and conducted a complete-case analysis.

### Outcomes and measures

Outcomes were obtained from standardized questionnaires administered to CHWs in 2021 and 2023, capturing exposure to and perception of violence in the communities where they live and work. For each type of violence, three dimensions were assessed: *perception* (recognition of violence as a relevant issue in their territory), *heard about/saw* (indirect exposure through events witnessed or reported by others), and *suffered* (direct personal [or immediately family] experience of violence). All outcomes represent community-level signals within CHWs' areas of practice. The questionnaire did not anchor the violence items to a fixed recall window; therefore, responses should not be interpreted as time-stamped events within a defined interval. Accordingly, violence was measured using a structured, non-diagnostic instrument designed to capture commonly reported and socially salient violent events in the territories where CHWs live and work. Rather than estimating incidence or prevalence of clinically defined violence, this approach aimed to approximate community-level exposure through CHWs’ direct experiences and sentinel awareness. This strategy aligns with population-based survey methods that use experiential and perceptual indicators as proxies for territorial violence dynamics.

### Ethical approvals

The data collection conducted in 2021 received approval from the Research Ethics Committee (CEP) of the State University of Ceará (UECE) with approval number 4.587.955, and the data collection in 2023 was approved by the CEP of IPADE with approval number 5.917.599.

All participants provided written Free and Informed Consent Form prior to participation. To safeguard privacy, questionnaires were administered in private space within CHWs respective health units, allowing participants to respond freely without the presence of supervisors or other third parties. Participation was voluntary, and participants were informed of their right to skip questions or withdraw from the study at any time without penalty. Data collection was conducted using a structured questionnaire rather than open-ended interviews. No interviews were interrupted due to participant distress. All participating CHWs had access to institutional mental health and psychosocial support services available through Brazil's Unified Health System primary care network.

The ethical and safety procedures adopted in this study were designed to protect confidentiality and minimize potential discomfort for participants. These procedures are consistent with key principles outlined in the WHO Ethical and Safety Recommendations for Research on Domestic Violence against Women (2001) and the updated WHO guidance for intervention research on violence against women (2016), particularly regarding voluntary participation, privacy during data collection, and the protection of both participants and research staff. By incorporating these principles, data collection aimed to create an environment that supported participant safety and facilitated open disclosure.

### Role of the funding source

This study was supported by multiple funding sources, including FUNCAP and Fiocruz-PMA in Brazil, and the Lemann Research Fundation, Harvard University, US. The funding bodies had no role in the data collection, analysis, or dissemination of the findings.

## Results

In our study, the CHWs live and work in the same communities, which means that their reports reflect this dual role. Male CHWs perceive more urban violence than female CHWs. In contrast, female CHWs perceive more domestic violence than male CHWs. Overall, the pattern of greater direct victimization among male (“suffered”) coexists with a distinct pattern in witnessed events (“heard about/saw”), providing insight into the social ecology of violence, highlighting a context in which male appear more directly exposed to urban forms of violence (assault, firearms, gangs), whereas female emerge as sentinels for domestic violence.

In 2021, the questionnaire was completed by 1942 CHWs (1599 female and 343 male). In 2023, the instrument was reapplied and answered by 1907 CHWs (1554 female and 353 male). The majority of CHWs were aged 40–60 years (70% [n = 1348] in 2021, and 73% [n = 1330] in 2023), self-identified as brown race (72% [n = 1395] in 2021, and 71% [n = 1359] in 2023), had a domestic partner (58% [n = 1133] in 2021, and 56% [n = 1077] in 2023), and had worked as a CHW for more than 10 years (82% [n = 1585] in 2021 and 88% [n = 1516] in 2023) (Table 1). There was no statistically significant association between gender and race (Cochran-Mantel-Haenszel χ^2^ = 2.90, df = 3; *p* = 0.41).

### Differences in the perception of violence by gender

When evaluating the types of violence most commonly perceived in the territories covered by the CHWs (Table 2), there was no consistent difference in overall perception between female and male CHWs. However, patterns emerged when violence was examined by type. For domestic violence, no difference between female and male working as CHWs was observed in 2021 (*p =* 0.50; female 48% [n = 737] *vs* male 46% [n = 152]), whereas in 2023, female reported higher perception than male (*p =* 0.041; 45% [n = 705] *vs* 39% [n = 139]). The Cochran-Mantel-Haenszel (CMH) test accounting for CHW gender and year, indicated a borderline overall difference (*p =* 0.06; CMH OR 1.18 [95% CI 1.00–1.40]). In contrast, urban violence was consistently reported more frequently by male in 2021 (*p =* 0.019; female 79% [n = 1212] *vs* male 85% [n = 280]) and 2023 (*p =* 0.008; 79% [n = 1223] *vs* 85% [n = 300]). The CMH test showed a significant gender-based difference across both years (*p <* 0.001; CMH OR 0.67 [0.53–0.83]). The Breslow–Day (BD) test showed no significant heterogeneity across strata for domestic violence (*p =* 0.34) or urban violence (*p =* 0.87), indicating that the odds ratios were homogeneous.

### Direct and indirect exposure to violence

Focusing on direct exposure (suffered), the data in Table 3 showed a consistent pattern of higher victimization among male CHWs across several public-space outcomes (such as assault, gang activity, or firearm-related incidents), with statistically significant differences in the 2021–2023 analysis. In 2021, female and male working as CHWs reported similar experiences of violence, with no statistically significant differences, except for rape, where more female CHWs reported having heard about/seen such events than males (*p =* 0.016; female 28% [n = 449] *vs* male 22% [n = 74]).

In contrast, in 2023, gender differences became more pronounced across multiple forms of violence. Male working as CHWs more frequently reported having heard about/seen assault (*p =* 0.014; female 81% [n = 1029] *vs* male 88% [n = 247]) and were more often direct victims of physical aggression (*p =* 0.027; female 35% [n = 384] *vs* male 42% [n = 104]), stabbing (*p =* 0.023; female 16% [n = 173] *vs* male 22% [n = 54]), non-lethal gunshot (*p <* 0.001; female 22% [n = 247] *vs* male 34% [n = 84]), and gang violence (*p <* 0.001; female 26% [n = 290] *vs* male 38% [n = 93]).

The CMH test indicated borderline differences for assault (*p =* 0.06) and gang violence (*p =* 0.06) in terms of having heard about or seen such events. However, significant differences were observed for direct exposure to violence, with male CHWs reporting higher rates of physical aggression (*p =* 0.023; CMH OR 0.80 [95% CI 0.67–0.97]), stabbing (*p =* 0.022; CMH OR 0.75 [95% CI 0.59–0.95]), and non-lethal gunshot (*p <* 0.001; CMH OR 0.71 [95% CI 0.58–0.87]). The BD test indicated homogeneity of odds ratios across strata.

Consistent with 2021 findings, in 2023 more female CHWs reported having heard about/seen rape compared with male CHWs (*p* = 0.019; female 39% [n = 499] *vs* male 31% [n = 91]), indicating that female consistently demonstrated greater awareness across both years. A significant gender-based difference across both years was confirmed by the CMH test (*p <* 0.001; CMH OR 1.40 [95% CI 1.15–1.70]), and the BD test (*p* = 0.93) again indicated homogeneity of odds ratios.

## Discussion

Our findings demonstrate marked gender-based differences in how CHWs in Northeastern Brazil perceived and were exposed to violence over 2021–2023, a timeframe encompassing the COVID-19 pandemic and its consequences. The findings underscore that although CHWs of both genders encounter violence in their professional territories, the nature, intensity, and recognition of such violence vary depending on the gender of the CHW. To deepen the interpretation of our findings, the following discussion is structured in two parts. First, we discuss the persistence patterns of urban violence in CHWs’ work contexts, highlighting how organized crime, socioeconomic marginalization, and weakened community bonds affect their ability to deliver care. Next, we discuss the evolving dynamics of domestic violence in the wake of the COVID-19 pandemic, we examine shifts in domestic violence over time, situating them in the broader context of post-pandemic social and economic pressures, evolving awareness, and disclosure practices.

Urban violence has emerged as a markedly more prevalent issue in Northeastern Brazil's state capitals large cities, which figures among the world's most violent metropolitan centers.^14^ PHC workers on the outskirts of large cities often operate in environments dominated by organized crime, gang activity, and severe socioeconomic deprivation, factors which drive higher levels of violence.^15^ CHWs working in “degraded and violent territories” struggle to deliver care, as constant exposure to territorial violence impedes home visits and outreach.^16^ Furthermore, urban violence disrupts the bond between CHWs and residents, restricting dialog and trust and ultimately hampering health promotion efforts.^17^ At the same time, our gender *versus* year analysis indicates that this urban/community signal did not depend on fluctuations in pandemic intensity: males consistently reported higher exposure in 2021 and 2023, suggesting that structural drivers in public space (weapons, gangs, contested territories) remain the dominant forces shaping their risk ecology. The implication is practical and immediate: even when social mobility and services normalize, territorial risk for field teams persists. Moreover, pandemic-related social disorganization and economic strain likely sustained and, in some contexts, worsened urban violence levels through 2023.^18^^,^^19^

In general, our comparison suggests that between 2021 and 2023, UV remained high with a consistent gender pattern, whereas DV showed a more salient post-pandemic signal among female in 2023, reflected in a borderline pooled effect. During the pandemic, particularly in 2021, CHWs’ ability to detect cases of violence was likely constrained by lockdowns and reduced face-to-face contact with the community. Indeed, CHWs across the Northeast region reported that pandemic-related restrictions hindered routine home visits and community engagement,^8^ likely allowing some cases of domestic abuse to go unnoticed. This post-pandemic rise in visibility aligns with broader global trends: numerous sources warned of a “shadow pandemic” of DV during COVID-19, as victims were trapped at home with their abusers.^20^ With the reopening of communities, a global surge in reports of violence against women and children was observed.^21^

This global context helps explain our local findings. During the strictest lockdown periods (such as in 2020 and early 2021), many cases of violence remained hidden or underreported due to mobility restrictions. Reports from Brazil reflect this reality: during the peak of isolation measures in 2020, police-recorded cases of domestic violence dropped by an average of 25% compared to 2019.^22^ This decline likely does not reflect a decrease in violence itself, but rather reduced access to reporting mechanisms, stemming from fear, lack of privacy to seek help or the suspension of routine home visits by CHWs and other state agents. As a result, the true magnitude of domestic violence may have been obscured during this period. Notably, communities in non-capital cities, where monitoring was already more difficult, reported a marked increase in the visibility of violence against women. This resurgence supports broader concerns that the lifting of restrictions would reveal a backlog of unreported or unnoticed abuse.^23^ This pattern mirrors national and international evidence indicating that violence trends during and after the pandemic primarily reflect shifts in visibility, reporting, and social conditions rather than direct causal effects of COVID-19.

Beyond increased visibility, our findings also highlight the challenges of recognizing and responding to normalized violence. The proportion of CHWs who perceived domestic violence as a relevant issue in their area was relatively low, especially when compared to other forms of violence. This may be due to a normalization effect: in many communities, certain abusive behaviors are so entrenched in household dynamics that they are not readily identified as violence by external observers or even by the victims themselves. This interpretation is supported by literature on primary healthcare in Brazil, which points to the invisibility of violence in routine services.^24^ Often, violence is neither reported nor addressed because it is seen as a private matter, and both healthcare workers and families may lack the training to recognize subtle forms of abuse.^24^ In this context, CHWs, whose main formal duties do not include identifying or addressing GBV may only report cases they directly witness or learn about, with subtler forms of abuse often remaining invisible. Without institutional guidance or mandates, more subtle forms of abuse such as psychological, economic, or less visible physical violence, are often perceived as part of everyday family dynamics and remain unaddressed.^24^ Rather than a failure on the part of CHWs, this gap reflects a structural omission in policy and training. Our findings, which reveal a high prevalence of both direct and vicarious violence reported by CHWs, underscore the urgent need for clearer health system guidance to support these frontline workers. Importantly, the workforce is predominantly female, reflecting global patterns that suggest CHWs’ lived experiences, including, in some cases, previous exposure to violence, may shape their sensitivity to gender-based issues.^25^ Despite their centrality to community engagement, CHWs are not formally trained or mandated to address domestic or sexual violence, leaving them without adequate referral mechanisms or protective protocols. This structural gap highlights a key policy priority: integrating CHWs into violence surveillance and support systems in ways that ensure both community safety and worker protection.

These findings must be interpreted in light of Brazil's structural and institutional context for community health work. CHWs are salaried municipal employees integrated into the Family Health Strategy (*Estratégia Saúde da Família*), a decentralized model within the SUS that assigns them to micro-areas where they also reside.^26^ This embeddedness fosters strong community ties but also exposes CHWs to the same vulnerabilities as the populations they serve. Despite their central role in health promotion, many face limited training, insufficient institutional support, and weak links to protection systems, particularly in areas marked by normalized violence. Compared to models in other countries, where CHWs often operate through NGO-based or volunteer systems with different scopes and protections, Brazil's approach ensures proximity but not necessarily protection or autonomy.^25^ The persistence of CHWs' exposure to violence reflects both territorial risks and systemic gaps in workforce support, shaping the gendered patterns and policy implications of our findings.

This embedded vulnerability is further illustrated in CHWs’ accounts of sexual violence. Our analysis of rape data among female CHWs reveals a deeply troubling trend. We observed a post-pandemic increase in awareness/sentinel surveillance, more pronounced among female. For rape heard about/saw, female reported consistently higher levels in 2021 (28.45% [n = 449] *vs* 22.02% [n = 74] among male; *p* = 0.016) and 2023 (38.74% [n = 499] *vs* 31.38% [n = 91]; *p* = 0.019), with a robust pooled effect (CMH OR 1.40 [95% CI 1.15–1.70]; p < 0.001; BD *p* = 0.928), suggesting greater detection and visibility of these events in day-to-day primary health care. Homogeneity across strata (Breslow–Day >0.05) reinforces that the effect is stable across years, indicating a replicable pattern rather than a spurious result from a single time point. This sharp increase suggests a growing vulnerability to extreme forms of violence in the post-pandemic period, particularly in urban areas with high crime rates. The northeast region of Brazil illustrates this vividly, with eight of its nine capitals ranked among the 50 most violent cities globally in 2024.^14^ In Fortaleza alone, more than 80% of CHWs reported being exposed to community violence, whether as victims or witnesses.^7^

Three of the four non-capital municipalities in our study lie within Ceará’s semi-arid Cariri region, approximately 600 km from the nearest state capitals, Fortaleza (Ceará State) and Recife (Pernambuco State). This territory is recognized as one of the highest-risk areas for women in the Northeast, with an average of six reports of GBV recorded daily in the region.^27^ Such elevated risk reflects both chronic social vulnerability and the impact of organized crime, exposing CHWs to persistent community violence and heightening their occupational hazards.^9^ The result may be a persistent underreporting of sexual violence: in Brazil, it is estimated that 90% of cases are never formally reported.^28^ This pervasive silence reflects wider trends in GBV in the country.

This study offers a unique and comprehensive view of violence as experienced and perceived by CHWs across municipalities in Northeastern Brazil, covering a critical temporal span from the height of the COVID-19 pandemic (2021) to its aftermath (2023). A major strength lies in the large, multicentric, and stratified sample. The dual time-point design enhances the ability to capture evolving trends in community violence and CHW perception, while the focus on both domestic and urban violence provides multidimensional insight. Nonetheless, some limitations must be acknowledged. A limitation of this study is that the violence items were not anchored to a fixed recall window. Accordingly, individual reports may reflect experiences accumulated across CHWs' tenure in the territories where they live and work, rather than incidents occurring within a clearly defined time interval. Gender was self-reported with response options “feminine”, “masculine”, and an open “other” category with free-text specification. Although an open “other” category was available in the questionnaire, no participants selected this option. Consequently, analyses were limited to female and male as reported, and do not allow further stratification beyond the categories observed in the data. Moreover, because no pre-pandemic (pre-2020) primary data were available, differences observed between 2021 and 2023 cannot be interpreted as causal effects attributable to COVID-19. Even so, the comparison between the 2021 and 2023 waves remains informative as a population-level assessment of changes in reported perceptions and experiences of violence among CHWs across two distinct phases of the pandemic trajectory, using the same sampling framework and instrument. Violence was measured through a structured self-report questionnaire focused on specific, highly recognizable events, which may undercapture less visible forms of violence, particularly psychological, economic, and institutional violence. This is especially relevant for GBV, where coercive control and emotional or financial abuse are often hidden and underreported. Nevertheless, although not designed to provide a clinical diagnosis, this approach enables systematic identification of recurrent, high-impact violent events affecting daily life and work in vulnerable territories, offering a broader view than homicide rates alone and reflecting violence as experienced and perceived by CHWs. The data are based on self-reports, which may be subject to recall or social desirability biases. Additionally, while the sample is representative of selected municipalities, the findings may not be generalizable to all regions of Brazil. It is important to emphasize that these findings do not exclusively reflect violence occurring ‘on the job’. Rather, they capture CHWs' broader lived reality, which includes their own direct victimization, violence affecting family members, and cases they witnessed or became aware of in their communities. This distinction is critical to interpret the dual vulnerability of CHWs as both health professionals and community members. Despite these limitations, the study significantly advances knowledge in an underexplored field and provides essential evidence to inform gender-sensitive and territorially responsive public health policies.

Our findings call for moving from broad policy statements to concrete actions. Table 4 outlines targeted measures, training, surveillance integration, referral pathways, and structural protections to strengthen CHWs' response to violence while safeguarding their wellbeing. Violence in CHWs' service areas is multifaceted and patterned by gender and type rather than locality alone. Between 2021 (pandemic) and 2023 (post-pandemic), the overall burden remained stable, but complementary gendered signals became clear: male more often reported urban/community violence and showed higher direct victimization in public-space outcomes while female showed a year-specific elevation in the perception of DV (evident in 2023) and consistently higher sentinel awareness of sexual violence. These findings offer actionable guidance for primary care while recognizing that CHWs’ reports are self-reported sentinel signals that should be interpreted with appropriate caution. Ultimately, ensuring the protection, recognition, and integration of CHWs within violence response systems is not only a matter of occupational safety, it is a strategic imperative for strengthening frontline public health in contexts of chronic insecurity and social vulnerability.

**Table 4:** Specific policy recommendations to support CHWs in addressing violence and protecting their wellbeing.

Domain	Recommendation	Rationale/expected impact
**Training**	Develop standardized training protocols on identifying, documenting, and responding to violence (domestic, sexual, community).	Enhances CHWs' capacity to recognize both overt and subtle forms of violence and respond sensitively.
**Surveillance**	Integrate CHWs' reports and observations into municipal and national violence surveillance systems.	Ensures early detection of trends, improves data accuracy, and informs targeted public policies.
**Referral mechanisms**	Establish clear, mapped, and operational referral pathways linking CHWs to social assistance, mental health care, women's protection services, legal aid, and law enforcement units. Ensure CHWs know whom to activate, when, and how, including emergency and after-hours escalation procedures.	Prevents cases from remaining isolated within communities; ensures victims receive timely and comprehensive care; reduces burden on CHWs who currently navigate referrals informally; promotes intersectoral collaboration at the service delivery level.
**Structural support**	Implement system-level protections for CHWs through: (i) national legislation recognizing violence exposure as an occupational risk; (ii) mandatory safety protocols enforced by health authorities; (iii) institutional accountability mechanisms for municipalities failing to safeguard CHWs; (iv) guaranteed access to psychosocial support and legal protection with dedicated funding; and (v) CHW representation in municipal security and intersectoral governance councils.	Addresses structural determinants of CHW vulnerability through binding policy instruments; creates enforceable obligations rather than voluntary measures; strengthens governance frameworks; ensures financial and institutional sustainability; promotes accountability from national to local levels; positions CHW safety as a system-level priority.

## Contributors

MRC, MCRT, MCC, AKY, and APGFVM conceptualized the study. MRC, MCRT, and APGFVM led the drafting of the manuscript. AG, MG, MCC, and AKY provided critical revisions to improve the manuscript. RSS, SFF, FDF, YPCCS, and APPM were responsible for data collection, and RSS and APGFVM verified and conducted the data analysis. All authors contributed to the revision process, reviewed the final manuscript, and approved it for submission.

## Data sharing statement

The data presented in this study are available upon reasonable request from the corresponding author. The data are not publicly available due to ethical restrictions.

## Editor note

The Lancet Group takes a neutral position with respect to territorial claims in published maps and institutional affiliations.

## Declaration of interests

The authors have no conflicts of interest in this work.
