# Supplementary material for: Domestic and urban violence faced by community health workers: a multidimensional analysis in vulnerable territories in northeastern Brazil during and after the COVID-19 pandemic
Source: Lancet Reg Health Am. 2026 Mar 11;57:101436. doi: 10.1016/j.lana.2026.101436 (PMC12995465; doi:10.1016/j.lana.2026.101436)
Supplement: Supplementary Material [file mmc1.pdf]

Domestic and Urban violence faced by community health workers: a multidimensional analysis in vulnerable territories in northeastern Brazil during and after the COVID-19 pandemic

Marcella R. Cardoso, PhD[1,2,3,4,†]; Maria Cecília Ramiro Talarico, MSc[4,†]; Roger Silva Sousa, PhD[5]; Sidney Feitosa Farias, PhD[5]; Franklin Delano Forte, PhD[6]; Yana Paula Coelho Correia Sampaio, MSc[7]; Ana Patrícia Pereira Morais, PhD[8]; Mary Greenwald, JD[1]; Marcia C. Castro, PhD[9]; Aisha K. Yousafzai, PhD[9]; Annekathryn Goodman, MD, MPH[1,2,3,‡]; Anya Pimentel Gomes Fernandes Vieira-Meyer, PhD[10,‡].

Supplementary Materials

List of content:

- Questionnaire (Original version in Portuguese)
- Questionnaire (Translated version in English)

Original Version in Portuguese:

QUESTIONÁRIO APLICADO AO AGENTE COMUNITÁRIO DE SAÚDE (ACS)

Olá, estamos felizes que tenha concordado em participar da presente pesquisa. Este questionário é dividido em várias partes e gostaríamos de iniciar fazendo algumas **perguntas pessoais.**

1. DADOS SOCIODEMOGRÁFICOS

1. Qual a sua idade: ______ anos

2. Gênero:
( ) Masculino ( ) Feminino ( ) Outro. Qual? ______________________________

3. Qual a Cidade onde mora?
( ) Crato
( ) Fortaleza
( ) João Pessoa
( ) Juazeiro do Norte
( ) Recife
( ) Sobral
( ) Teresina

4. Qual o seu Estado Civil?
( ) Solteira(o)

( ) Casada(o)/união estável
( ) Viúvo (a)
( ) Separado (a) /divorciado (a)

5. Qual sua escolaridade?
( ) Analfabeto
( ) Ensino Fundamental Incompleto
( ) Ensino Fundamental Completo
( ) Ensino Médio Incompleto
( ) Ensino Médio Completo
( ) Ensino Superior Incompleto
( ) Ensino Superior Completo

2. DADOS PROFISSIOGRÁFICOS

17. Há quanto tempo (anos) você trabalha como ACS na Estratégia Saúde da Família?
______ anos

Gostaríamos agora de fazer algumas perguntas **sobre a violência na comunidade** em que você mora e trabalha.

3. EXPOSIÇÃO DO ACS À VIOLÊNCIA COMUNITÁRIA

3.1 A violência está presente na comunidade onde você atua?
( ) Não ( ) Sim

3.2 Quais são os tipos, situações ou formas mais comuns de violência no seu território de atuação? (**pode marcar mais de uma)**
[ ] Violência doméstica (Por exemplo: Entre marido e mulher; familiares, etc.);
[ ] Violência urbana/comunitária (Por exemplo: assalto, briga de gangue, etc.);
[ ] Violência institucional (Por exemplo: Dificuldade de acesso [no posto, escola, CRAS,outros]);
[ ] Outros. Quais? ____

Gostaríamos agora de fazer algumas perguntas sobre **sua experiência com a violência Urbana/Violência na comunidade** em que você mora e trabalha **assim como na sua casa**.

4. DADOS SOBRE A EXPOSIÇÃO DO ACS A VIOLÊNCIA URBANA/COMUNITÁRIA

Das formas de violência listadas abaixo, relacione aquelas que VOCÊ OU UM MEMBRO DE SUA FAMÍLIA já **VIU/SOUBE** ou que já **ACONTECEU COM VOCÊ/MEMBRO DE SUA FAMÍLIA**, no ambiente da comunidade em que vocês vivem:

Tipo de violência	Viu/soube	Aconteceu
4.1. Agressão Física	( ) Não ( ) Sim	( ) Não ( ) Sim
4.2. Assalto	( ) Não ( ) Sim	( ) Não ( ) Sim
4.3. Esfaqueamento	( ) Não ( ) Sim	( ) Não ( ) Sim

4.4. Tiro não fatal	( ) Não ( ) Sim	( ) Não ( ) Sim
4.5. Tiro fatal	( ) Não ( ) Sim	( ) Não ( ) Sim
4.6. Estupro	( ) Não ( ) Sim	( ) Não ( ) Sim
4.7. Violência relacionadas as gangues/facções	( ) Não ( ) Sim	( ) Não ( ) Sim
4.8. Outro tipo de violência	( ) Não ( ) Sim	( ) Não ( ) Sim

Translated Version in English:

QUESTIONNAIRE FOR COMMUNITY HEALTH WORKERS (CHWs)

Hello, we are pleased that you have agreed to participate in this research. This questionnaire is divided into several parts, and we would like to begin by asking some **personal questions**.

1. SOCIODEMOGRAPHIC DATA

1. **What is your age?** ______ years old
2. **Gender:**
   ( ) Male ( ) Female ( ) Other. Please specify: ________________________________
3. **What city do you live in?**
   ( ) Crato
   ( ) Fortaleza
   ( ) João Pessoa
   ( ) Juazeiro do Norte
   ( ) Recife
   ( ) Sobral
   ( ) Teresina
4. **What is your marital status?**
   ( ) Single
   ( ) Married/in a stable union
   ( ) Widowed
   ( ) Separated/divorced
5. **What is your educational level?**
   ( ) Illiterate
   ( ) Incomplete Elementary School
   ( ) Completed Elementary School
   ( ) Incomplete High School
   ( ) Completed High School
   ( ) Incomplete Higher Education
   ( ) Completed Higher Education

2. OCCUPATIONAL DATA

17. **How long (in years) have you worked as a CHW in the Family Health Strategy?**
    ______ years

We would now like to ask you some questions **about violence in the community** where you live and work.

3. CHW EXPOSURE TO COMMUNITY VIOLENCE

3.1 Is violence present in the community where you work?
( ) No ( ) Yes

3.2 What are the most common types, situations, or forms of violence in your area of work?
(You may select more than one)
[ ] Domestic violence (e.g., between spouses, among family members, etc.)
[ ] Urban/community violence (e.g., assault, gang fights, etc.)
[ ] Institutional violence (e.g., lack of access to health centers, schools, social services)
[ ] Other. Please specify: ____

We would now like to ask you some questions about **your experience with urban/community violence** in your home and the community **where you live and work**.

4. DATA ON CHW EXPOSURE TO URBAN/COMMUNITY VIOLENCE

For the types of violence listed below, indicate those that YOU OR A FAMILY MEMBER have either **witnessed/heard about** or that have **happened to YOU/A FAMILY MEMBER** in your community:

Type of Violence	Witnessed/Heard About	Happened
4.1. Physical assault	( ) No ( ) Yes	( ) No ( ) Yes
4.2. Robbery	( ) No ( ) Yes	( ) No ( ) Yes
4.3. Stabbing	( ) No ( ) Yes	( ) No ( ) Yes
4.4. Non-fatal shooting	( ) No ( ) Yes	( ) No ( ) Yes
4.5. Fatal shooting	( ) No ( ) Yes	( ) No ( ) Yes
4.6. Rape	( ) No ( ) Yes	( ) No ( ) Yes
4.7. Gang/faction-related violence	( ) No ( ) Yes	( ) No ( ) Yes
4.8. Other type of violence	( ) No ( ) Yes	( ) No ( ) Yes
